# CPAP IMPACT: a protocol for a randomised trial of bubble continuous positive airway pressure versus standard care for high-risk children with severe pneumonia using adaptive design methods

**DOI:** 10.1136/bmjresp-2017-000195

**Published:** 2017-06-30

**Authors:** Andrew G Smith, Michelle Eckerle, Tisungane Mvalo, Brian Weir, Francis Martinson, Alfred Chalira, Norman Lufesi, Innocent Mofolo, Mina Hosseinipour, Eric D McCollum

**Affiliations:** 1 Paediatric Critical Care Medicine, University of Utah, Salt Lake City, Utah, USA; 2 Division of Emergency Medicine, Cincinnati Children's Hospital Medical Center, Cincinnati, Ohio, USA; 3 UNC Malawi Project, Lilongwe, Malawi; 4 Johns Hopkins University Bloomberg School of Public Health, Baltimore, Maryland, USA; 5 Malawi Ministry of Heath, Lilongwe, Malawi; 6 Division of Infectious Disease, University of North Carolina at Chapel Hill, Chapel Hill, North Carolina, USA; 7 Eudowood Division of Pediatric Respiratory Sciences, Johns Hopkins School of Medicine, Baltimore, Maryland, USA

**Keywords:** Pneumonia, Paediatric Lung Disease

## Abstract

**Introduction:**

Pneumonia is a leading cause of mortality among children in low-resource settings. Mortality is greatest among children with high-risk conditions including HIV infection or exposure, severe malnutrition and/or severe hypoxaemia. WHO treatment recommendations include low-flow oxygen for children with severe pneumonia. Bubble continuous positive airway pressure (bCPAP) is a non-invasive support modality that provides positive end-expiratory pressure and oxygen. bCPAP is effective in the treatment of neonates in low-resource settings; its efficacy is unknown for high-risk children with severe pneumonia in low-resource settings.

**Methods and analysis:**

CPAP IMPACT is a randomised clinical trial comparing bCPAP to low-flow oxygen in the treatment of severe pneumonia among high-risk children 1–59 months of age. High-risk children are stratified into two subgroups: (1) HIV infection or exposure and/or severe malnutrition; (2) severe hypoxaemia. The trial is being conducted in a Malawi district hospital and will enrol 900 participants. The primary outcome is in-hospital mortality rate of children treated with standard care as compared with bCPAP.

**Ethics and dissemination:**

CPAP IMPACT has approval from the Institutional Review Boards of all investigators. An urgent need exists to determine whether bCPAP decreases mortality among high-risk children with severe pneumonia to inform resource utilisation in low-resource settings.

**Trial registration number:**

NCT02484183; Pre-results.

## Introduction

Despite laudable reductions in global childhood mortality rates, pneumonia remains the second most frequent killer of children <5 years old worldwide.^1 2^ Nearly 1 million children died from pneumonia in 2013, with greater than half of deaths in Africa.^2^ In the southern African country of Malawi, which has a high prevalence of severe malnutrition and HIV infection, pneumonia is a major cause of paediatric mortality.^3^ In low-resource settings like Malawi, current treatment for severe pneumonia consists of hospital admission, antibiotics and low-flow oxygen.^4^ Even with improved adherence to these treatments, the case fatality rate for children in low-middle income countries with severe pneumonia complicated by severe malnutrition, HIV infection, HIV exposure or severe hypoxaemia remains elevated.^5–9^

Bubble continuous positive airway pressure (bCPAP) is a relatively inexpensive mode of non-invasive ventilation. bCPAP treats hypoxaemic and hypercarbic respiratory failure by expanding lungs towards optimal functional residual capacity, which can improve gas exchange and work of breathing by optimising ventilation/perfusion matching, improving lung compliance and reducing airway resistance. It has been successfully used in resource-rich settings for decades in the treatment of respiratory distress syndrome in neonates. Several trials have demonstrated the efficacy and feasibility of bCPAP in treating neonates with respiratory distress in low-resource settings.^10^

bCPAP has been proposed as a treatment modality for children with severe pneumonia in low-resource settings, including children with high-risk conditions such as HIV infection, HIV exposure without infection (HUE), severe malnutrition and severe hypoxaemia.^11–14^ However, evidence of bCPAP’s effectiveness in children with severe pneumonia in low-resource settings is lacking.

Our group has documented favourable severe pneumonia outcomes in Malawian children using bCPAP in case series and reports.^15–17^ A recent randomised controlled trial conducted in Bangladesh demonstrated that children with severe hypoxaemic pneumonia had decreased mortality among those treated with bCPAP compared with standard care of low-flow oxygen.^8^ While this study’s results provide critical proof of concept, its generalisability to low-resource settings with higher pneumonia case fatality rates remains unclear. For example, the study was conducted in an intensive care unit although the majority of children aged 1–59 months with severe pneumonia are treated in district hospitals that lack intensive care.^12^ Additionally, the Bangladesh study was terminated at interim analysis due to perceived efficacy which has led some to argue that the results are difficult to interpret.^18^

No studies evaluating bCPAP among African children with severe pneumonia and high-risk conditions exist to date. Children with HIV infection, HUE, severe malnutrition and/or hypoxaemia are at greater risk of pneumonia mortality than other children and are also represented disproportionally among child pneumonia deaths globally.^3 12 19–21^ Identifying bCPAP’s impact on children with these high-risk conditions is important because of their high prevalence in low-resource African settings.^3 21–23^ Further reductions in global child pneumonia mortality will require interventions that successfully target these groups.

In addition to determining bCPAP’s effectiveness in pneumonia treatment, specific implementation challenges must also be addressed. While bCPAP may be effective in certain settings like tertiary referral hospitals, wider implementation of bCPAP to the district hospital level where most children receive hospital care may have substantial challenges. For example, bCPAP devices, while not as costly as invasive methods of ventilation, are generally more expensive than traditional low-flow oxygen nasal cannula delivery. bCPAP also requires greater patient-provider ratios of clinicians with specialised training to ensure accurate, continuous pressure delivery. Identifying the subset of patients most likely to benefit from bCPAP will assist with resource allocation, particularly where bCPAP access is limited.

Prior to wider investment and implementation of bCPAP for non-neonatal pneumonia in low-resource settings, it is necessary to understand whether bCPAP use confers a mortality benefit among high-risk children. Therefore, we are conducting a prospective randomised trial comparing bCPAP to low-flow oxygen among an African paediatric population with endemic HIV and a high prevalence of severe malnutrition to answer this question. The paper describes our research methods and rationale and outlines our efforts to manage two goals: (1) a pragmatic study design that allows for generalisable results; (2) a study that maintains the rigours of a randomised controlled trial.

## Methods and analysis

### Study design and setting

The study is an unblinded, randomised, superiority trial with parallel assignment and a 1:1 allocation ratio. We used an adaptive design strategy (see ‘Sample size and power using adaptive design methods’ in the Methods and analysis section).

We are conducting the trial in a district hospital. District hospitals are where the majority of children in low-resource settings are treated for severe pneumonia.^12^ The study is designed to be representative of everyday care in order to optimise the generalisability of our results, while maintaining the scientific rigour of a trial.

Salima District Hospital (SDH) in Malawi is the project site. SDH is a 250-bed government facility; the paediatric department admits approximately 7000 children annually. The site was chosen, with input from the Malawi Ministry of Health, since SDH is similar to other Malawi district hospitals in terms of clinical staff and laboratory and radiology capabilities.

### Recruitment and enrolment

All children admitted to the paediatric ward between the ages of 1 and 59 months are first triaged by study staff. Study staff triage patients 24 hours/day. Children identified with WHO severe pneumonia are then consented and screened for eligibility by study staff.

#### Inclusion/exclusion criteria

**Figure 1:**
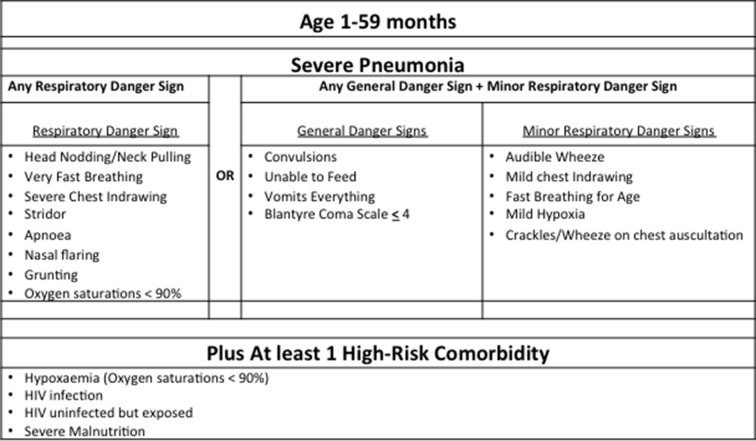
Inclusion criteria.

Children aged 1–59 months are enrolled if their caregiver provides written informed consent, they meet WHO-defined severe pneumonia criteria (figure 1) and have one of three high-risk conditions: severe hypoxaemia, HIV infection or exposure, and/or severe malnutrition.^4^

We defined severe figure 6yhypoxaemia as a peripheral oxyhaemoglobin saturation (S_p_O_2_) <90% as measured non-invasively with a pulse oximeter (Masimo Rad5 with LNOP® Y-I Multisite wrap sensor). HIV infection is a positive HIV DNA PCR for children <12 months or a positive HIV antibody test for children ≥12 months.^4^ In accordance with Malawi guidelines, children <24 months old who are uninfected but have an HIV-infected mother, irrespective of breast feeding status, are considered HUE.^4^ Severe malnutrition is a weight for height of ≤3 SD from the median and/or bilateral pedal oedema.^4^ In this study, children with a mid-upper arm circumference (MUAC) <115 mm are also classified as having severe malnutrition.^4^ We included children with a MUAC <115 mm, including those between 1 and 6 months, because they have been found to have an increased mortality risk when hospitalised for pneumonia.^9 24^

Exclusion criteria are limited to previous study enrolment or psychosocial conditions that would interfere with the study.

#### Randomisation and blinding

Simple 1:1 randomisation is conducted at enrolment using sequentially numbered sealed opaque envelopes. Due to the nature of the bCPAP intervention this study is unblinded for study staff and caregivers.

### Intervention

Children randomised to the trial’s study arm are initiated onto bCPAP with oxygen, while those randomised to the trial’s control arm are initiated onto low-flow oxygen only. All other treatment is the same for both arms and follows WHO and Malawi guidelines for severe pneumonia and other conditions.^4^ Children randomised to the control arm and non-study patients do not receive bCPAP as this is not standard of care in Malawi. In addition, invasive ventilation is not available as part of routine care in Malawi, and is therefore not available for study patients who are in either arm.

#### Equipment and support

bCPAP is delivered using the *Fisher & Paykel Bubble CPAP* system. This system is a validated non-invasive ventilator device that provides warmed humidification and pressure control. bCPAP flow is driven using *Newlife Intensity Airsep* oxygen concentrators capable of delivering 10 liters per minute (LPM) and 90%–97% fractional inspired oxygen concentration to the bCPAP system via oxygen tubing. In our experience, we need oxygen flows of between 6 and 8 LPM to achieve 5–8 cmH_2_0 bCPAP pressure.

**Figure 2:**
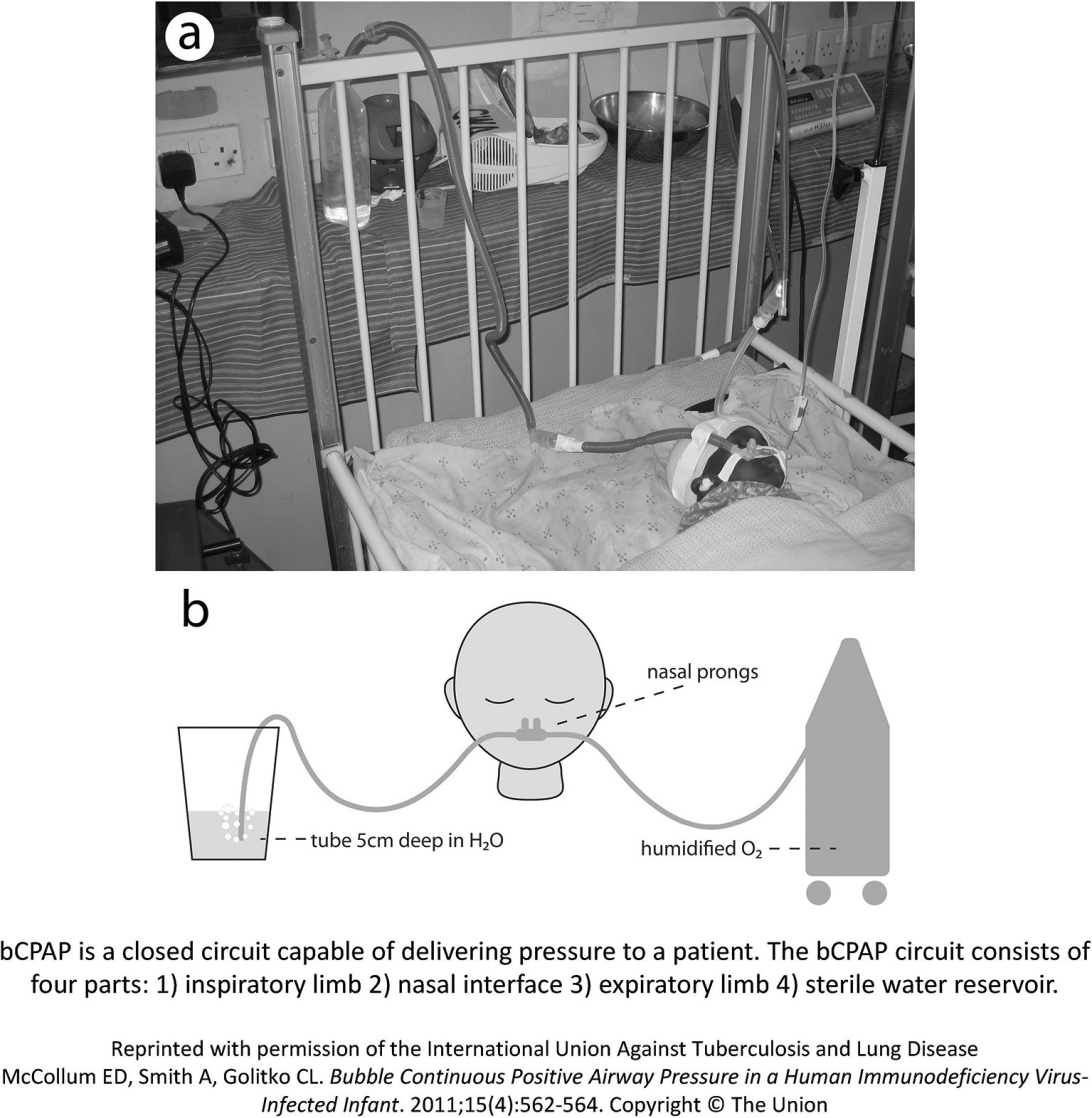
bCPAP design. bCPAP, bubble continuous positive airway pressure.

bCPAP is a closed circuit capable of delivering pressure to a patient (see figure 2). The bCPAP circuit consists of four parts: (1) inspiratory limb, (2) nasal interface, (3) expiratory limb and (4) sterile water reservoir. The inspiratory limb connects to the nasal interface, which is placed on the child.

We use two types of nasal interfaces based on the patient’s size: (1) nasal prongs and (2) unvented nasal masks. We elected to not use full-face masks for delivering bCPAP given the increased safety risks of aspiration and secretions with full-face masks.

The expiratory limb connects the patient’s nasal interface to a reservoir of sterile water, and is inserted into the reservoir of water at a depth equivalent to the desired pressure (eg, 5 cm depth is equivalent to 5 cm H_2_0 pressure).

bCPAP is a closed system. However, bCPAP pressure can be lost at the nasal interface if the seal is broken or via the child’s mouth. We confirm the desired bCPAP pressure with a digital manometer at least twice per day and after all pressure or flow adjustments.

We use the *Newlife Intensity Airsep* oxygen concentrator with nasal prongs to deliver low-flow oxygen for children enrolled in the control arm. The *Airsep* concentrato*r* is recommended by the WHO for use in low-resource settings and delivers up to 10 LPM. We use the *Caire Sure Flow* to split oxygen flow from a single concentrator for multiple patients. With the *Airsep* concentrator, the *Caire Sure Flow* can provide 2 LPM of oxygen for up to five patients.

A back-up generator guarantees continuous electrical supply.

#### Respiratory support protocol

Children triaged with severe pneumonia are immediately placed on low-flow oxygen as is standard care for SDH. Children remain on low-flow oxygen during consent, screening for eligibility and randomisation. Because the study enrols 24 hours/day, this process generally takes less than 60 min.

Children randomised to the control arm are continued on low-flow oxygen. Those children randomised to the bCPAP arm are initiated on bCPAP. Both bCPAP and low-flow oxygen initiation parameters are age dependent.

Enrolled children are reviewed every 4–6 hours until discharge from the hospital. Additional evaluations are conducted as needed based on clinical course.

For children on bCPAP, those between the ages of 30 and 59 days are started on a pressure level of 7 cmH_2_0; children >2 months old are started on 8 cmH_2_0. For those randomised to low-flow oxygen, children 30–59 days old on low-flow oxygen are started on 0.5 LPM; children >2 months old are started on 2 LPM, per WHO guidelines.^4^

**Figure 3:**
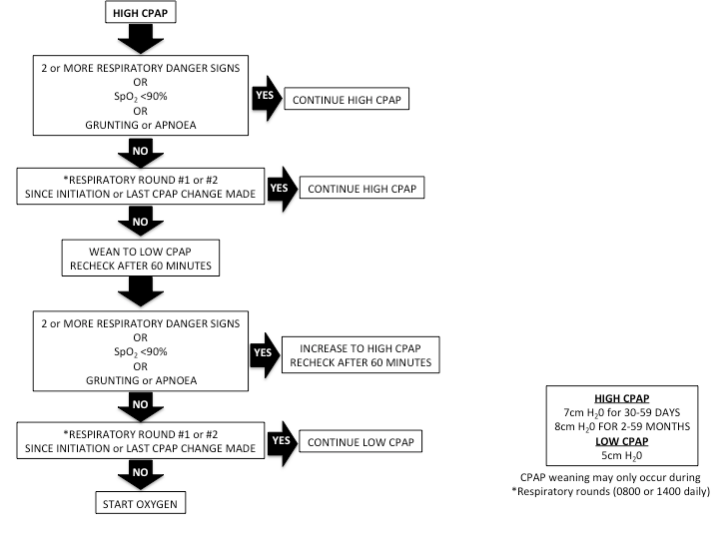
Weaning protocol for bCPAP. bCPAP, bubble continuous positive airway pressure.

**Figure 4:**
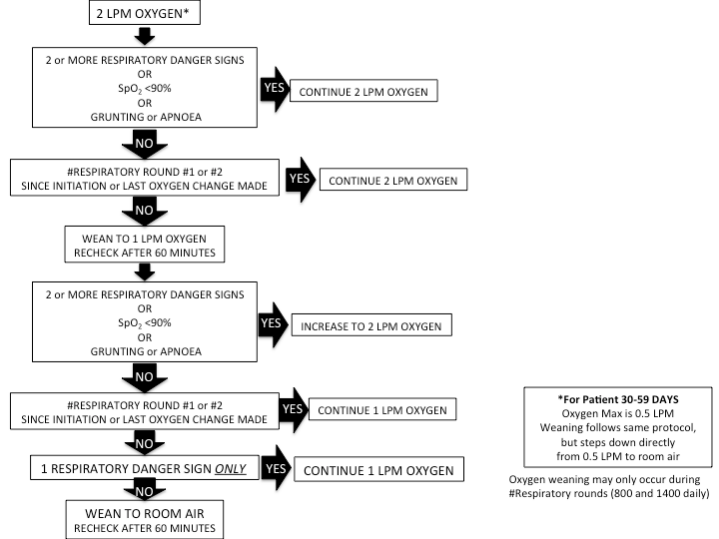
Weaning protocol for low-flow oxygen. LPM, liters per minute.

Children are weaned in a stepwise process based on vital signs and danger signs during the daytime respiratory status rounds. Children need to be without multiple respiratory danger signs or severe hypoxaemia for at least 24 hours, prior to any incremental respiratory support weaning (bCPAP or oxygen). Figures 3 and 4 outline our weaning protocols for bCPAP and low-flow oxygen.

#### Antibiotics

All enrolled children are started on parenteral benzylpenicillin and gentamicin at standard WHO doses and schedule.^4^ HIV-infected and HUE children <12 months old are also started on enteral cotrimoxazole and prednisone for presumptive *Pneumocystis jirovecii* infection.^4^ Patients who remain with fever in combination with persistent respiratory or general danger signs or who develop a new respiratory or general danger sign after 3 days of benzylpenicillin and gentamicin are switched to ceftriaxone.

Patients with altered mental status at any time are administered meningitic doses of parenteral ceftriaxone. Any child unable to be weaned off of respiratory support after 5 days of treatment is transitioned to ceftriaxone.

Children >12 months old with HIV infection or HUE are started on cotrimoxazole and prednisone after 3 days of benzylpenicillin and gentamicin if they persist with fever, in combination with persistent respiratory or general danger signs, or have a new respiratory or general danger sign.

Patients receive at least 5 days of parenteral benzylpenicillin and gentamicin. Children switched to ceftriaxone receive a total of at least 5 days of ceftriaxone. All children initiated on cotrimoxazole complete a 21-day course and a 5-day course of prednisone.

#### Feeding

Children with any general danger sign, 3 or more respiratory danger signs, or apnoea or grunting in isolation are not allowed to eat by mouth. Instead, these children receive a nasal gastric tube for feeding of expressed breast milk or formula every 2 hours at a standardised amount calculated to provide appropriate calories and fluid. Children with severe malnutrition receive nasal gastric feeds following the WHO protocol for severe malnutrition.^4^

Children able to eat by mouth and without severe malnutrition receive standard hospital food or food brought by their family. Children with severe malnutrition and able to eat by mouth receive malnutrition feeds per WHO recommendations.^4^

#### Additional treatments

All children are evaluated for malaria using a rapid blood test. Patients who are malaria positive are treated with a 3-day course of artesunate monotherapy per Malawi guidelines.

Children are also evaluated for anaemia via bedside haemoglobin measurement. Children without severe malnutrition are transfused with packed red blood cells for a haemoglobin level <6 mg/dL; children with severe malnutrition are transfused for a haemoglobin level <4 mg/dL. Blood product availability is typical of Malawi district hospitals and is often unreliable.

Children with dehydration and shock are treated following the recent WHO guidelines for fluid resuscitation.^13^ Children with altered mental status have their glucose measured and are treated with dextrose if hypoglycaemic per WHO recommended thresholds.^4^

### Follow-up

We contact all guardians of enrolled children by phone 30 days following discharge and collect information on the child’s current condition.

### Sample size and power using adaptive design methods

This study will accrue 900 children (450 children per study arm) with pneumonia and high-risk comorbidities. Mortality assumptions for this study were derived from published literature and programmatic data from Malawi.

We estimate true mortality rates of 14.7% with standard care and 6.1% for bCPAP cases. With a 95% follow-up rate and α=0.05, the power (1 – β) is estimated to be 99%. Assuming mortality of 14.7% in the standard care arm, the sample is sufficient to reject the null hypothesis with 80% probability if the mortality rate in the bCPAP arm is 8.7% or lower.

**Figure 5:**
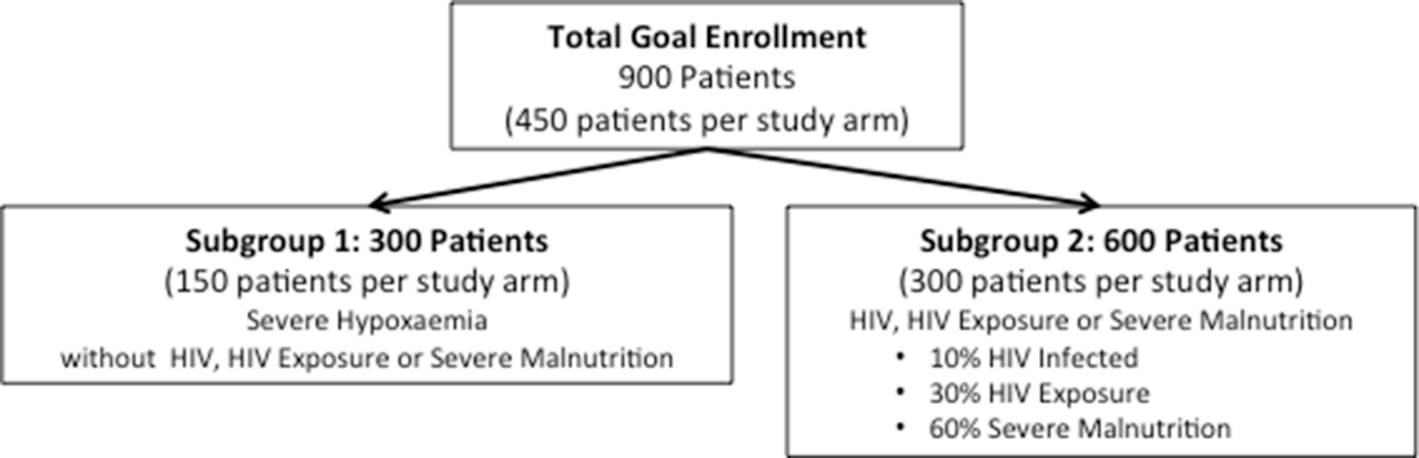
Projected enrolment.

The study cohort is further divided into two subgroups of high-risk conditions (figure 5). Distributions for subgroups were reassessed using adaptive design methods from data collected during the first 10 months of the study. All effect estimates were determined *a priori*.

- Severe hypoxaemia without comorbidity subgroup: 300 (150 per arm) children with severe hypoxaemia but without HIV infection, HUE or severe malnutrition.
  - Power analysis subgroup 1: we estimate 13% mortality for standard care and 4% for bCPAP cases.^8^
  - With 95% follow-up, equal allocation to the standard care and bCPAP conditions, and the hypothesised true mortality rates described, these analyses are powered at 80%.
- Comorbidity subgroup: 600 children (300 per arm) with HIV infection, HUE or severe malnutrition.
  - Power analysis subgroup 2: we estimate 15.6% mortality for standard care and 7.1% for bCPAP cases based on relative contributions by each subgroup extrapolated from observational data. The observational data reveal approximately:
    - 10% of children are infected with HIV, with assumed standard care mortality of 30% and bCPAP mortality of 14%.^7 9^
    - 30% of children have HUE, with assumed standard care mortality of 12% and bCPAP mortality of 5%.^7^
    - 60% of children are severely malnourished (HIV uninfected and HIV unexposed), with assumed standard care mortality of 15% and bCPAP mortality of 7%.^5^
  - With 95% follow-up, equal allocation to the standard care and bCPAP conditions, and the hypothesised true morality rates described, these analyses are powered at 89%. Assuming mortality of 15.6% in the standard care arm, the sample is sufficient to reject the null hypothesis with 80% probability if the mortality rate in the bCPAP arm is 8.3% or lower.

### Data collection, quality assurance and data management

All study staff are certified in Good Clinical Practice and were trained on study protocols and data collection. Staff complete refresher-training courses every 4–6 months and receive regular supervision.

Trial data are collected directly into a secure REDCap (Research Electronic Data Capture) database using encrypted tablets. REDCap is a secure, web-based application designed to support data capture for research studies. Paper case report forms are used for back-up when necessary.

### Statistical methodology and analysis

Logistic regression will be used in the primary and interim outcome analyses. Hospital pneumonia outcome (pneumonia cure vs died) will be the primary endpoint; study arm (bCPAP vs standard care) will be the treatment variable in primary and interim outcome analyses.

We will employ the O’Brien-Fleming stopping rule to address any inflated type II error stemming from multiple comparisons.^25^ p Values (and Z-score cut-offs) for two-sided tests used in the first interim, second interim and final analyses will be .0006 (3.438), .0151 (2.431) and .0471 (1.985), respectively.^26^

If any significant imbalance between groups on baseline covariates is present then results of logistic regression analyses controlling for these variables will also be done. If missing data do not appear to be missing completely at random, multiple imputation methods will be used to model the missinginess mechanism based on observed data and to evaluate whether the study findings vary when accounting for missinginess.

Secondary analyses will examine whether treatment effect varies by baseline patient characteristics. As with the primary analyses, hospital pneumonia cure versus died before hospital pneumonia cure will be the outcome in logistic regression with independent variables including group assignment, patient characteristic and an interaction between group assignment and patient characteristic.

Separate models will be run for each patient characteristic of interest, including primary risk category—comorbidity (HIV infection or HUE, and/or severe acute malnutrition) and severe hypoxaemia. We will also examine whether severe hypoxaemia modifies the effect of treatment for children with a comorbidity through the use of interaction terms.

## Ethics and dissemination

### Ethical approval and consent

The study received approval from the Johns Hopkins Institutional Review Board and the Malawi National Health Sciences Research Committee. Written informed consent is obtained from all eligible children’s guardians prior to enrolment.

### Patient safety

Study staff are present in the hospital at all times (ie, 24 hours/day and 7 days/week). All adverse events are reported immediately to a study investigator and documented in a separate adverse event form. Death and withdrawal from the study unexpectedly are considered adverse events. The Institutional Review Boards and Data Safety and Monitoring Board (DSMB) review cumulative safety and study data after 30% enrolment and 60% enrolment and ad hoc as is necessary. The DSMB reports are shared with both the funding agency and investigators.

### Dissemination

We plan to disseminate results of CPAP IMPACT in peer-reviewed journals and international conferences, targeting those involved in the clinical care of children in low-resource settings as well as those who develop and advise on policy and protocols in those settings, especially the Malawi Ministry of Health, Acute Respiratory Infection Control Programme. We also plan to provide this data set on an open access platform at the conclusion of the trial. The trial is registered at clinicaltrials.gov (NCT02484183).

## Discussion

The following discussion outlines our efforts to manage the following two goals: (1) a pragmatic study design that allows for applicable and generalisable results to real-world, non-study settings; (2) a study that maintains the rigours of a randomised controlled trial protocol.

### Efforts towards generalisable results

#### All children with comorbidity are started on oxygen independent of SpO_2_

We chose to initiate all enrolled children with a comorbidity (HIV infection or HUE, and/or severe malnutrition), regardless of SpO_2_, on oxygen by either low flow or bCPAP for four main reasons. First, children without severe hypoxaemia but with severe pneumonia and a comorbidity still have elevated mortality, suggesting that severe hypoxaemia should not be the only indication for bCPAP in this group of children. In most resource-rich settings, the observation of an acute illness with increased work of breathing in selected higher risk children can itself be an indication for bCPAP, since the continuous positive pressure delivered can ease respiratory distress by improving lung compliance and reducing airway resistance. Second, pulse oximetry is not widely available in Malawi; severe pneumonia, in the absence of oximetry, is a clinical indication for oxygen treatment.^4^ Third, there is evidence from Malawi that children with pneumonia and moderate hypoxaemia (SpO_2 _90%–92%) have a greater risk of hospital mortality, calling into question the currently recommended Sp0_2_ threshold of <90% for oxygen initiation.^9^ Lastly, bCPAP requires 6–8 LPM flow to maintain pressure, and this flow is delivered using an oxygen concentrator. We do not expect that district hospitals will have the technology to titrate the fractional oxygen content of the delivered flow required to maintain bCPAP pressure. In addition to an oxygen concentrator and pulse oximetry, titrating fractional oxygen content of bCPAP flow would require delivery of room-air flow and a separate oxygen analyser.

#### Children enrolled in our trial are diagnosed with severe pneumonia based on clinical criteria

Given our goal is to make the trial results as generalisable as possible, we choose to use clinical diagnosis of WHO severe pneumonia alone as inclusion criteria. While chest radiographs may add improved specificity to the clinical diagnosis of severe pneumonia, the majority of low-resource settings do not have access to radiographs and are diagnosed with severe pneumonia based on WHO criteria alone.^27^

In addition, chest radiographs alone are an imperfect reference standard to diagnose pneumonia and are not routinely recommended for pneumonia diagnosis by the WHO.^4 28^ Instead, chest radiographs are recommended mainly for children who fail first-line pneumonia treatment.^4^ In the recently completed multicountry Pneumonia Etiology for Child Health study, approximately 45% of children with chest indrawing or danger signs had a normal chest radiograph (Fancourt N, personal communication).

#### We limit bCPAP pressures to 8 cmH_2_0 (children aged 2–59 months) and 7 cmH_2_0 (children aged 1–2 months)

An important risk of higher bCPAP pressures is a pneumothorax. Higher bCPAP pressures are commonly used in resource-rich settings with advanced, continuous monitoring that allows for immediate detection of a pneumothorax. In a low-resource setting, with limited monitoring, we determined *a priori* that we would limit bCPAP pressures to pressures safely used in previously reported observational studies.^16 17^ Notably, two blinded paediatric intensivists independently reviewed this protocol and agreed with this approach prior to study implementation. Future work could examine whether higher bCPAP pressures are feasible, effective and safe in routine care settings with limited supervision and safety measures.

#### We are enrolling children with severe pneumonia but without comorbidity (HIV infection or exposure, and/or severe malnutrition) and severe hypoxaemia into a parallel observational substudy

As already detailed in this work, we used existing mortality evidence to define our higher risk severe pneumonia cases as children with HIV infection, HUE, severe malnutrition or severe hypoxaemia. However, there may be other at-risk groups of children not enrolled into the trial. Therefore, to better understand whether CPAP IMPACT’s enrolment criteria have captured those at greatest mortality risk, and to better understand whether there are additional key mortality risk factors in those children not included in CPAP IMPACT, we are following until hospital outcome those children with severe pneumonia but without a comorbidity or severe hypoxaemia. Children enrolled into the observational substudy are initially evaluated by our study staff and determined to be without comorbidity. Haemoglobin levels and malaria status are collected, standard WHO severe pneumonia treatment is prescribed and then the child is transferred into the care of SDH government staff. Hospital outcomes are recorded. This comprehensive study design should provide a deeper understanding of severe pneumonia cases at a Malawian district hospital while also validating whether CPAP IMPACT has appropriately targeted children at the greatest mortality risk.

### Efforts towards rigorous protocol

#### We are using the *Fisher & Paykel Bubble CPAP* system for this study’s bCPAP device

While such a system is standard of care in most resource-rich settings, a hybrid, locally designed bCPAP system is more commonly used in low-resource settings.^15 16^ Previously we have used locally designed systems, but for this study we instead chose to use the commercially available Fisher & Paykel system.

Our previous work identified that if flow is not warmed and humidified, secretions can harden and cause nasal obstruction.^15^ The Fisher & Paykel system delivers warmed, humidified flow. In addition, we determined that, for this initial trial, using a validated commercial device would increase the precision of bCPAP pressure to the patient.

#### We are using dedicated study staff to care for children enrolled in the trial

We determined that a dedicated study staff is necessary to assure that bCPAP is reliably implemented, protocols are followed and data are accurately collected. The dedicated study personnel were recruited from the public sector and have comparable skill levels as government staff.

#### We have provided the study site with a back-up generator

Electricity can be intermittent in Salima, Malawi. Our study installed a back-up electrical generator to assure uninterrupted power to the bCPAP devices and oxygen concentrators. While using a generator may decrease generalisability to district hospitals without back-up power supply, assuring uninterrupted power decreases the likelihood that electricity interruptions will become a confounding variable in evaluating bCPAP’s efficacy.

## Conclusion

To our knowledge, this study is the first to examine the impact of bCPAP on mortality among children with severe pneumonia and high-risk conditions in a district hospital setting representative of where the majority of children in low-resource settings receive treatment for severe pneumonia. Our study’s results should inform policymakers and clinicians as they make decisions of whether to implement bCPAP as a pneumonia treatment for their populations, especially in African settings with endemic HIV, severe malnutrition and hypoxaemia.
